# Prompt and precise identification of various sources of infection in response to the prevention of malaria re-establishment in China

**DOI:** 10.1186/s40249-022-00968-y

**Published:** 2022-04-18

**Authors:** Jianhai Yin, He Yan, Mei Li

**Affiliations:** National Institute of Parasitic Diseases, Chinese Center for Disease Control and Prevention (Chinese Center for Tropical Diseases Research), NHC Key Laboratory of Parasite and Vector Biology, WHO Collaborating Center for Tropical Diseases, National Center for International Research on Tropical Diseases, Shanghai, 200025 China

**Keywords:** Malaria, Re-establishment, Diagnosis, Laboratory competency, Quality control

## Abstract

Prompt and precise diagnosis of patients is an essential component of malaria control and elimination strategies, it is even more vital for the prevention of malaria re-establishment in the post elimination phase. After eliminating malaria in China, the strategy for prevention of malaria re-establishment was updated in a timely manner from the elimination strategy focusing on each case/focus to the prevention of re-establishment focusing on timely identification of the source of infection. However, there are numerous challenges, such as the persistent large number of imported malaria cases, the long-term threat of border malaria, unknown levels of asymptomatic infections and *Plasmodium falciparum* HRP2/3 gene deletions, and the continuous spreading of antimalarial drug resistance. Meanwhile, the detection capacity also need to be further improved to meet the timely detection of all sources of infection, otherwise it is bound to occur introduced malaria cases and malaria re-establishment in the presence of malaria vector mosquitoes. Therefore, it is necessary to continuously strengthen the malaria detection competency at all levels, promote the research and development on the malaria parasitological testing technologies, thus improving the timely detection of various sources of infection, and preventing the re-establishment of malaria.

**Graphical abstract:**

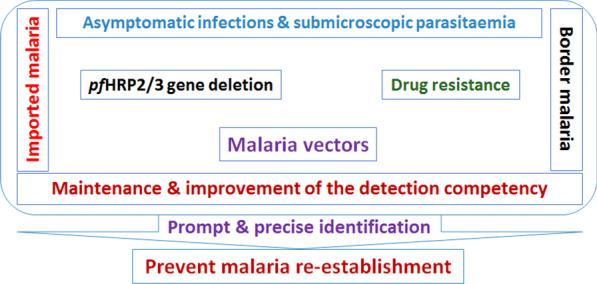

## Background

China officially achieved the elimination of malaria on June 30, 2021 [1]. But globally, there are all kinds of good and bad stories in the fight against malaria. Fortunately, there has been a historic breakthrough in the application of malaria vaccines funded from the Global Alliance for Vaccines and Immunization [2], the World Health Organization (WHO) recommended that children living in sub-Saharan Africa and other areas with moderate to high falciparum malaria transmission be vaccinated against RTS,S/AS01 vaccine [3], although this vaccine can only reduce the infection rate by 40% and the incidence of severe malaria in children by about 30%. Unfortunately, malaria drug resistance has been one of the most pressing threats to the global public health community, more serious is the independent emergence and local transmission of artemisinin-resistant *Plasmodium falciparum* in Africa found in samples from Uganda from 2017 to 2019 [4]. Therefore, there is still a long way to go to achieve global eradication of malaria.

With the advancement of the global malaria control process, more and more countries have entered the pre-elimination stage or the malaria elimination stage. The WHO launched the E-2020 initiative in 2017, and a total of 21 malaria-endemic countries are on track to reduce indigenous malaria cases to zero by 2020 [5]. In 2018, Paraguay became the first E-2020 country to be certified malaria-free by WHO, and later Algeria, El Salvador and China were also certified malaria free [5, 6]. Now is the time for the E-2025 initiative, and the WHO has listed another 25 countries that are on track to achieve malaria elimination by 2025 [7]. The failure to successfully implement the E-2020 initiative may have been due to the impacts of the coronavirus disease 2019 (COVID-19) pandemic. After the outbreak of the COVID-19, the WHO predicted that malaria in sub-Saharan Africa would increase significantly, based on the impact of the pandemic on the implementation of malaria prevention and control measures and the availability of antimalarial treatment. This has been effectively confirmed in the World Malaria Report 2021[8], the estimated number of cases in 87 endemic countries in the world reached 241 million, and the estimated number of deaths reached 627,000 in 2020. Compared with 2019, the number of malaria cases and deaths have increased significantly, and about two-thirds of the new deaths were related to the disruption of medical services for malaria due to the COVID-19 pandemic. Sub-Saharan Africa remains the worst affected region by malaria, where 80% of malaria deaths were in children under five. Therefore, the WHO recommends that countries strengthen primary health care and provide better health services including malaria prevention, diagnosis and treatment.

Prompt, precise diagnosis and treatment of patients with an effective medicine is an essential component of malaria control and elimination strategies [9]. Confirmatory malaria diagnosis is even more vital in areas with successful malaria control programmes, where the malaria incidence is declining and the likelihood that malaria is the cause of fever is reduced. Meanwhile, parasitological testing is the only way to diagnose malaria accurately in febrile patients. Moreover, accurate malaria diagnosis can also greatly improve the reliability of surveillance data, allowing public health officials to more accurately describe and assess the state of malaria transmission, and help policy makers and investors allocate resources effectively.

Following the elimination of malaria in China, the strategy for prevention of malaria re-establishment was updated in a timely manner, from the elimination strategy focusing on each case/focus to the prevention of re-establishment focusing on timely identification of the source of infection. However, there are still several practical challenges. Here, we highlight the different sources of malaria infection and the malaria parasite testing competency that currently exist in China, and thus reminding everyone of the challenges faced in preventing malaria re-establishment.

## Multiple sources of malaria infection

Although indigenous malaria has been eliminated in China, she still faces the threat of multiple sources of malaria infection. Once malaria cases cannot be detected and managed effectively, this will be detrimental to the consolidation of malaria elimination achievements.

### High imported cases burden

China has been under tremendous pressure from imported malaria over the years [10]. Before the COVID-19 pandemic, there were about 3,000 cases every year, mainly from Africa and Southeast Asia [10]. Five *Plasmodium* species were reported, with *P. falciparum* being the most prevalent, followed by *P. vivax*, and the proportion of *P. ovale* has been on the rise for several years [10]. These imported malaria cases were not only large in quantity, but also widely distributed across China [11]. They were reported in all provinces of the country, and most of them were in historically malaria-endemic provinces. In addition, they were reported throughout the year, and June and July was the cumulative peak period, especially in historically malaria-endemic areas [11].

### Challenging border malaria

Regarding border malaria in China, it was always said that "Whether China can eliminate malaria depends on Yunnan, and whether Yunnan can eliminate malaria depends on the border". Fortunately, the last indigenous malaria case was reported in Yunnan Province in 2016, which was also the last indigenous case in China [12]. But there are two types of border malaria that must be monitored. The first is imported cases. The border of Yunnan Province is 4060 km long and has 25 counties border Myanmar, Laos and Vietnam. Since 2017, these border counties were still facing huge pressure of imported cases, particularly in counties on the China-Myanmar border [13]. The second is malaria infections caused by positive *Anopheles* mosquitoes that cross the border and cause human infection within China, and has been found at a Chinese construction site on the China-Myanmar border. It is particularly important to detect such patients at the border in time to block secondary transmission after the elimination of malaria in China, because the mosquitoes can come and go freely across borders without a passport.

### Undetermined asymptomatic infections/submicroscopic parasitaemia

The imported cases mentioned above usually refer to those with clinical manifestations, and patients often seek medical treatment on their own initiative to be detected and reported, making them the focus of malaria prevention and treatment. However, numerous studies have described asymptomatic malaria infections detected by molecular methods, but missed by traditional diagnostics in all intensities of malaria transmission, and these infected people rarely seek medical treatment, or cannot be identified when seeking medical treatment, resulting in underreporting [14]. Furthermore, asymptomatic infections contribute far more to the malaria reservoir than previously thought, and symptomatic malaria cases are only the tip of the iceberg of malaria infection sources [14]. This may be one of the main factors for the continuous spread of malaria.

The detection of asymptomatic infections depends largely on the testing methods. Nucleic acid detection technologies such as polymerase chain reaction (PCR) have become an ideal means to monitor and detect asymptomatic infections, with a detection limit far lower than that of conventional microscopy and rapid diagnostic tests (RDT). For instance, malaria infection rates detected by microscopy were on average only half of those detected by PCR and even lower [15]. The positive rate of microscopy is closely related to the prevalence of malaria, which is higher when the prevalence is high, and lower when the prevalence is low. Meanwhile, all *Plasmodium* species have asymptomatic infection, not only *P. falciparum* and *P. vivax* [16], but also *P. ovale* and *P. malariae* [17]. In some Chinese border counties on the China-Myanmar border, asymptomatic infections of *P. falciparum* and *P. vivax* were found, and the levels of asymptomatic infections detected by different PCR methods were also different [18].

### Increasing HRP2/3 gene deletions

RDT is widely used in the rapid screening of malaria cases due to its simple operation and short time required to complete the detection. However, the species of *Plasmodium* in different regions are different, and the antigens expressed by different *Plasmodium* vary from region to region. At present, histidine-rich protein 2 (HRP2), lactate dehydrogenase and aldolase are used to detect malaria parasite antigens in various combinations. While HRP2 is produced only by *P. falciparum* and used to specifically detect *P. falciparum*, and commercial products that target other antigens are not yet available. However, more and more countries around the world, especially African countries, have reported the gene deletion of *P. falciparum* HRP2/3 (WHO Malaria Threats Map), which makes the RDT detection reagent based on this protein unable to detect *P. falciparum* very well, and this ratio can even be as high as 50% reported in some studies in the Horn of Africa region (WHO Malaria Threats Map). Meanwhile, Africa is the main source of imported cases in China, which virtually increases the risk of these cases that are not detected by *Pf*HRP2 RDTs. In addition, up to 15.4% of HRP2 deletions in *P. falciparum* isolates from different infection sources were found in one survey conducted in Yunnan Province [19]. Therefore, the WHO Malaria Policy Advisory Group called on the world to attach great importance to the high incidence of *Pf*HRP2/3 gene deletions in Africa and beyond, and to detect these infections in a timely and accurate manner [20].

### Spreading antimalarial drug resistance

Antimalarial drugs are one of the main means of malaria control and play an important role in the fight against malaria. However, *Plasmodium* has developed resistance to different drugs one after another since the 1960s and 1970s, and the resistance has spread rapidly around the world [21], especially the generation and spread of artemisinin-resistant strains has become a huge challenge for global malaria eradication, particularly the emergence of independent artemisinin-resistant strains on the African continent [4]. At the same time, in addition to the resistance of *P. vivax* to pyrimethamine and sulfa doxine, different degrees of resistance to chloroquine and mefloquine have also been reported in Asia, America and other regions [22]. Hence, it is important to monitor the antimalarial drugs resistance levels in real time, particularly in *P. falciparum* and *P. vivax* which are the main species imported into China. If patients cannot be cured in a timely and effective manner, they are bound to become potential sources of infection.

In China, in vivo and in vitro assays and resistance gene detection methods are used to monitor the drug susceptibility of different antimalarial drugs, especially artemisinin drugs to *P. falciparum* and chloroquine to *P. vivax*, according to the WHO's recommendations. It was found that the sensitivity of *P. falciparum* to artemisinin drugs has decreased in the border area of Yunnan, but no clear resistance has been found [23–25]. Meanwhile, *P. vivax* was still sensitive to chloroquine [26]. These results strongly guaranteed the effective treatment of cases suffered from these two types of malaria species, which was essential in achieving malaria elimination in China. However, high point mutations in different antimalarial resistance genes were reported in the imported malaria cases from different regions into China in recent years [27–29]. Therefore, strengthening the establishment of an antimalarial resistance surveillance network to better monitor the imported *Plasmodium* resistance should be one of the key tasks after the elimination of malaria in China.

## Insufficient laboratory testing competency for malaria parasites

The detection of each malaria case mentioned above in a timely and accurate manner is important for China to eliminate malaria and prevent re-establishment. In the laboratory, malaria is diagnosed using different techniques, e.g. conventional microscopic diagnosis by staining thin and thick peripheral blood smears, and molecular diagnostic methods, such as PCR.

### Microscopy competency needs to be further improved

China has a group of high-level malaria microscopists, including a total of 19 microscopists obtained a level 1 certificate, 15 obtained a level 2 certificate, 7 obtained a level 3 certificate, and 6 obtained a level 4 certificate, by the end of 2019 [30]. Not only have they played a key role in the training and maintenance of the national malaria microscopy competency for malaria elimination, they will also play an important role in preventing re-establishment. However, even among experts with WHO credentials, their capacity for parasite counting on blood smears needs to be further improved [31]. Furthermore, it was reported that malaria cases mainly went to county and prefectural medical institutions for medical treatment, but only about 75% of the total cases could be diagnosed as malaria at the first visit, and the accuracy rates detected by medical and health institutions at different levels were also different, and around 90% accuracy was at the county and prefectural levels, and relatively rare *Plasmodium* species such as *P. malariae*, *P. ovale* and *P. knowlesi* were significantly more likely to be misdiagnosed [32]. In addition, China has held nine consecutive national competitions on parasitic disease prevention and control skills since 2011, and the overall competency of malaria parasite species identification and parasite counting by microscopy still needs to be further improved [33].

### Research and development of new diagnostic technologies needs to be strengthened

Microscopy of stained blood smears remains the standard method of malaria diagnosis in most parts of the malaria-endemic world, but it is experience dependent and cannot reliably distinguish *Plasmodium* species, resulting into difficulties with basing treatment decisions on microscopic results. Nucleic acid detection technologies such as PCR have become an ideal means to detect and identify *Plasmodium* species with higher sensitivity and specificity than that of conventional microscopy and RDTs. To date, the China Malaria Diagnosis Reference Laboratory Network has been established based on the institutes of parasitic diseases and centers for disease control and prevention (CDC) at national and provincial levels since the beginning of the national malaria elimination campaign [34], and one national laboratory and 25 provincial laboratories have joined [30]. These laboratories must have the ability to detect *Plasmodium* nucleic acid and carry out nucleic acid review for each reported case. Moreover, tertiary general hospitals, infectious disease hospitals, CDCs at all levels, and at least one county-level hospital in the county were required to have the competency of nucleic acid testing by the end of September 2020, and all secondary general hospitals also had this competency by the end of 2020, to respond effectively to the COVID-19 pandemic [35]. It provides a good platform for nucleic acid detection of other pathogens including malaria parasites. However, even if nucleic acid detection has been written into the diagnostic criteria of Diagnosis of Malaria (WS259-2015), and there are many commercial malaria parasite nucleic acid detection kits and different methods used in the CDCs, but none of them have been registered and approved by the China Food and Drug Administration and cannot be used in clinics routinely.

### Quality control needs to be continuously enhanced

The pathogenic microorganism test must ensure the accuracy of the test results and provide an effective reference for the treatment of clinical diseases. However, it is usually affected by a variety of factors in the routine practices, resulting in deviations in the test results. Thus, it is necessary to carry out quality control of pathogenic microorganism testing. In terms of quality control of *Plasmodium* detection, the WHO has corresponding quality control plans for malaria microscopy, RDT and nucleic acid detection respectively. China has also been participating in the relevant quality control activities of the WHO, especially in malaria microscopy and nucleic acid testing, and the performance is significantly better than the global average [36]. At the same time, China has also carried out similar quality control activities of malaria microscopy and nucleic acids testing in provincial laboratories in accordance with the WHO's quality control plan [34], which effectively guaranteed the detection quality of malaria parasites in China.

## Risks of malaria re-establishment

Malaria is a life-threatening but preventable and curable disease caused by *Plasmodium* parasites, which are transmitted to people through the bites of infected female *Anopheles* mosquitoes. China will likely see a resurgence in malaria cases if there is significant importation of malaria cases under the condition that the distribution of the malaria vector *Anopheles* mosquitoes, although it has been eliminated in the defined areas. Similar cases have been reported in China and abroad. For example, EU countries basically achieved malaria elimination in the 1970s, but there are lot of imported cases every year, mainly in France, Germany, Italy, the Netherlands, Spain and the United Kingdom [37]. More importantly, there are also *Anopheles* mosquito vectors that can effectively transmit malaria in these countries, especially the three types of *Anopheles atroparvus*, *Anopheles labranchiae*, and *Anopheles sacharovi* [38]. Unfortunately, malaria re-establishment outbreaks have been successively reported in Greece, Italy, Russia and other European countries [39–41]. Coincidentally, introduced cases have also occurred in Dandong of Liaoning province [42], Longhui of Hunan province [43], and Tengchong of Yunnan province [44] in China, which deserve attention post elimination.

## Conclusions

In conclusion, although China has successfully eliminated malaria, it still faces numerous challenges, especially the persistent large number of imported malaria cases, the long-term threat of border malaria, unknown levels of asymptomatic infections and HRP2/3 gene deletions, and the continuous spreading of antimalarial drug resistance. If the detection capacity cannot meet the timely detection of these sources of infection, it is bound to occur introduced malaria cases and malaria re-establishment, in the case of suitable distribution of malaria vector mosquitoes. Therefore, it is necessary to continue to maintain and strengthen the surveillance and response of imported malaria, improve the detection and diagnosis competency of malaria parasites of professionals in medical and health institutions at all levels through targeted training. Moreover, the diagnostic tools should be introduced into the clinic as soon as possible through continuous research and development of malaria parasite testing technologies, thus improving the timely detection of various sources of infection with a good quality control system, and preventing the re-establishment of malaria.

## Data Availability

Not applicable.

## Abbreviations

WHO: World Health Organization
COVID-19: Coronavirus disease 2019
PCR: Polymerase chain reaction
RDT: Rapid diagnostic tests
HRP2: Histidine-rich protein 2
CDC: Center for disease control and prevention
